# Direct Observations of Ordered Nanoporosity in Deprotonated 2-(Acetoacetoxy)ethyl Methacrylate (AAEMA^–^) Polymer: Preliminary Results

**DOI:** 10.3390/polym16010058

**Published:** 2023-12-23

**Authors:** Ambra M. Fiore, Saverio Fiore, F. Javier Huertas, Piero Mastrorilli

**Affiliations:** 1 Department of Chemistry, University of Bari “Aldo Moro”, 70125 Bari, Italy; 2 Institute of Methodologies for Environmental Analysis, National Research Council of Italy (IMAA-CNR), Tito Scalo, 85050 Potenza, Italy; saverio.fiore@cnr.it; 3 Instituto Andaluz de Ciencias de la Tierra (IACT, CSIC), 18100 Armilla, Granada, Spain; javier.huertas@csic.es; 4 DICATECh Department, Polytechnic University of Bari, 70125 Bari, Italy; piero.mastrorilli@poliba.it

**Keywords:** 2-(acetoacetoxy)ethyl methacrylate, AAEMA, microscopic investigations, ordered nanoporosity, proto-crystallinity

## Abstract

Polymers based on 2-(acetoacetoxy)ethyl methacrylate, charged with iron or sodium, were thermally heated at 150 °C. Both polymers were studied and characterized by SEM, TEM, STEM microscopy and SAEDF techniques. The morphological investigation revealed that, upon heating, both polymers were endowed with microholes, sometimes perfectly ordered, whose dimensions varied from 4–5 nm to approximately 500 nm. In the case of an Fe-containing copolymer, unexpectedly, iron did not fill in the cavities, thus implying that it was “dispersed” in the polymeric matrix. Electronic microdiffraction documented that both polymers exhibited a proto-crystallinity, likely induced by thermal heating.

## 1. Introduction

Polymers made of porous organic materials have experienced a raised level of interest in research areas thanks to their ability to combine the properties of porous and polymeric materials. The captivating possibility of designing materials based on their use has significantly increased the interest of the scientific community. In fact, one of the most relevant advantages is linked to the possibility of using these materials for their large surface area and their well-defined porosity [1]. Due to their easy processability and tuneable properties, porous polymers find applications in various fields, such as catalysis, sensing, drug delivery and environmental remediation. However, it is important to note that the specific processability of a porous polymer may depend on its composition, porosity and so on [2]. Besides, some of them can be treated with solvents, without modifying or destroying the porosity [3]. Such a property is characteristic for this material. For instance, certain natural porous materials like zeolites or metal–organic frameworks can also exhibit good processability depending on their synthesis method and properties. Another focus aspect is related to the careful design of the synthetic route of organic polymers. Researchers can tailor the properties of organic polymers, including their porosity and processability, to suit specific applications. These synthetic advancements play a vital role in expanding the range of porous materials available and enhancing their overall utility across various industries and technologies. A high number of chemical functionalities in organic polymers refers to the presence of multiple different reactive groups or chemical moieties within the polymer structure. These functionalities can be varied, allowing the polymer to interact with other molecules or materials and enabling a wide range of applications [4]. The introduction of specific chemical functionalities into the polymer structure allows for tailored interactions with external stimuli, leading to reversible changes in the pore structure. This behavior opens a wide range of applications in various fields. Polymeric frameworks, especially those used in porous materials, are often composed of light elements. The use of light elements offers several advantages and particular properties that are desirable for their intended applications [5]. Porous polymers can be designed to have high surface areas and tailored pore structures; in fact, they are excellent candidates for gas storage/separation [6,7,8,9], for controlled drug release [10] or as catalysts for their intrinsic catalytic activity [11]. Moreover, they can serve as support materials for catalytic nanoparticles or sensor elements [12]. Certain porous polymers can be thermally converted into nanostructured carbon materials, such as carbon nanotubes [13]. High-value applications of porous organic polymers have indeed driven a significant emphasis on the development of reliable methods for their preparation. As the research in this field continues to advance, the development of reliable methods for their synthesis will remain a central focus. The growing understanding of these materials, combined with innovative synthesis approaches, holds the promise of unlocking even more exciting and impactful applications in the future.

In a recent study aimed at synthesizing hematite nanoparticles from a polymer supported iron(III) complex [14], scanning electron microscopy (SEM) observations highlighted that the polymer occurred as “microporous” grains. However, it was unclear whether the Fe-nanoparticles (Fe-NPs) grew within the polymer, or if they filled in the cavities occurring on its surface. These are two aspects that needed to be clarified since acetoacetoxyethyl methacrylate (HAAEMA) has been extensively investigated in more than five-hundred patents targeting different applications such as nanoparticles [15], biocompatible cement [16] and dental resins [17], water treatment [18] or catalysts [19]. The interest in using HAAEMA as a comonomer is related to the presence of a β-ketoester functionality that critically improves the material properties; HAAEMA-based copolymers are more polar and therefore present enhanced adhesion properties and use a monomer without the presence of a hydroxyl group that could increase the viscosity of the reaction medium. The broad and versatile properties of HAAEMA have made it a compound of wide interest and applications [20,21]. To the best of our knowledge, no encompassing morphological study of HAAEMA copolymers has been reported in the literature thus far. The evaluation of morphology and structural order is of crucial importance for the synthesis of well-defined materials and/or to optimize the synthesis of polymer-supported nanoparticles. To investigate such an important physical characteristic of HAAEMA copolymers, we performed a detailed microscopic study using scanning and transmission electron microscopy (SEM-TEM).

## 2. Materials and Methods

Fe(AAEMA)_3_ was obtained as reported in the literature [22]. Sigma Aldrich chemical and reagents (St. Louis, MO, USA) were used The copolymer of Fe(AAEMA)_3_ (named Fe-POL) was obtained by copolymerization of the iron(III) AAEMA^–^ complex with N,N-dimethylacrylamide (DMAA) and N,N′-methylenebisacrylamide (MBAA) in N,N-dimethylformamide (DMF), following the procedure described in reference [22]. The NaAAEMA copolymer (named Na-POL) was obtained by copolymerization of NaAAEMA with DMAA and MBAA in DMF. HAAEMA (0.80 g) was dissolved in 10 mL of ethanol with NaOH (0.15 g); then MBAA (0.14 g), DMAA (2.5 g) and DMF (1.5 mL) were added. The mixture was heated at 50 °C for 24 h. The obtained Na-POL was washed with acetone and diethyl ether. Solid Fe-POL and Na-POL were both heated at 150 °C for 24 h to ensure removal of DMF.

Morphological observations and chemical analyses were performed using a high-resolution field emission scanning electron microscope (FESEM, Zeiss Supra 40; Carl Zeiss, Oberkochen, Germany) equipped with an energy dispersive X-ray spectrometer (EDS, Oxford Inca Energy 350; High Wycombe, England). The material was gently crushed using an agate mortar to obtain particles. A few milligrams of sample were dispersed in ultrapure water, and a drop of the dispersion was placed on a 12 mm Al stub covered by a carbon sticky table. The samples were then carbon coated. The TEM study was performed using a Thermo-Fisher Scientific TALOS F200X microscope (Waltham, MA, USA) operated at 200 kV, equipped with a Schottky field emission gun, and an EDX-type Super X with four detectors for analytical electron microscopy (AEM). The AEM spectra were collected in STEM mode (scanning TEM) using a high-angle annular dark-field (HAADF) on restrict areas. High-resolution images were obtained from ultra-thin sections. The samples were embedded in epoxy resin and cut with an ultramicrotome. The thickness of the section was approximately 200 nm.

## 3. Results and Discussion

**Figure 1:**
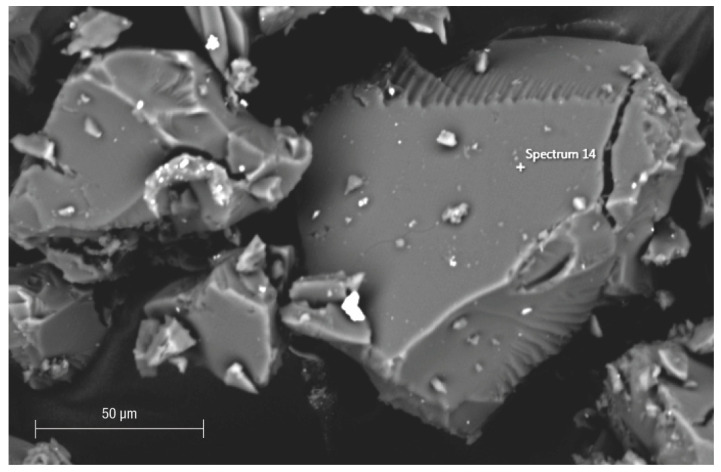
SEM image (backscattered electron detector) of compact grains of Fe-POL resembling glass shards. The chemical composition (by EDS) does not differ from that of porous grains.

Morphological observations showed that, according to the previous study [14], Fe-POL grains usually have a spongy surface; however, compact grains resembling glass shards were also observed (Figure 1).

**Figure 2:**
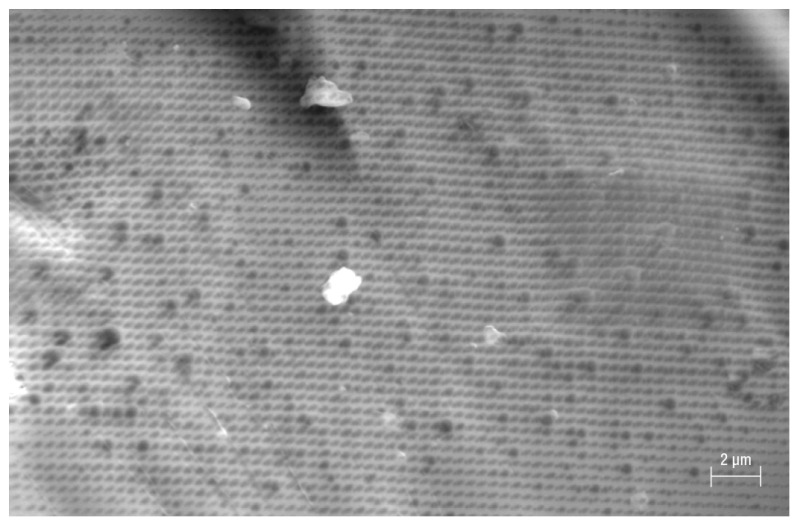
Nano and microholes on an Fe-POL grain (FESEM, SE detector). Nanoholes, perfectly ordered in two directions, have an elliptical outline (axes: 4–20 nm), but there are sometimes also circular ones. The larger holes always have a circular shape.

**Figure 3:**
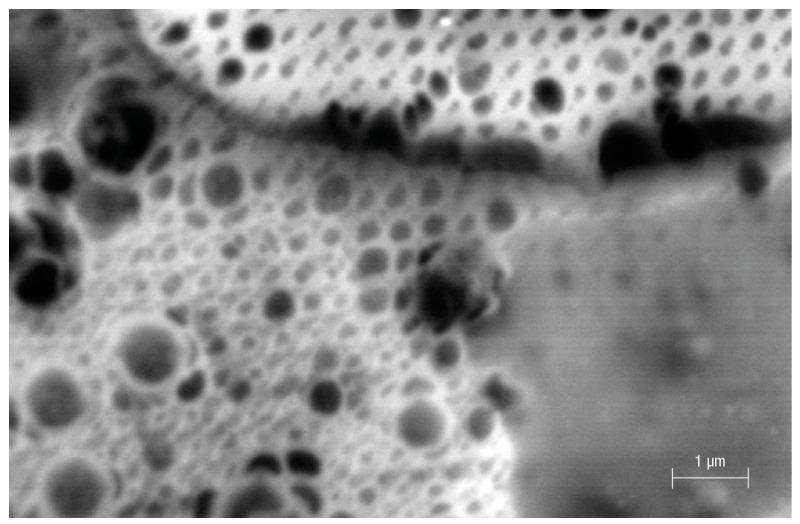
Surface of Fe-POL obtained using conventional SEM (W filament) and a backscattered electron detector. The size diameter of the smallest pores is approximately 10 nm. (see Figure 4).

**Figure 4:**
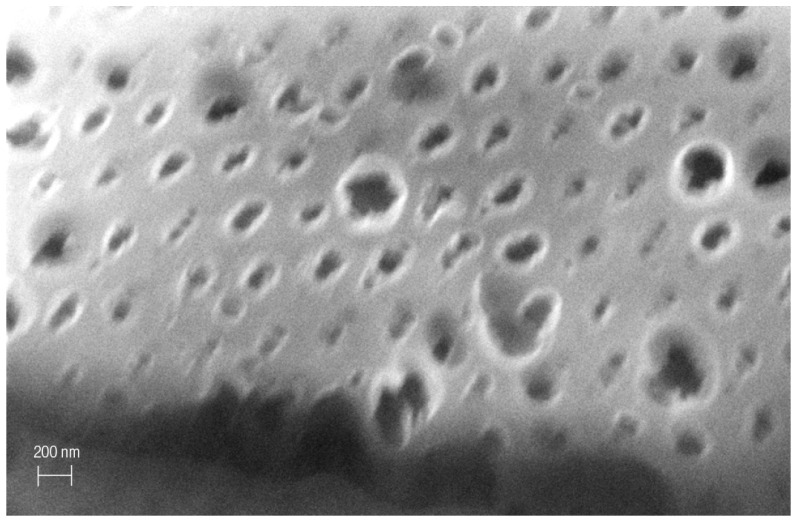
Secondary electron image of Fe-POL. The holes have variable dimensions and irregular outlines which suggests that their formation is not due to outgassing.

One grain of Fe-POL, whose dimension was approximately 1 mm, attracted attention because its surface exhibited a great number of holes (Figure 2). Their sizes were variable, from a few nanometres to more than one micrometre. The large “holes” had a circular outline and were randomly distributed, whereas the smallest ones had elliptical (axes: 4–20 nm) outlines and were perfectly aligned in two directions, thus resembling a grid. To exclude an instrumental artifact, the same sample stub was observed under a conventional SEM (tungsten cathode) using secondary and backscattered electron detectors. Other grains had exhibited several aligned nanoholes (elliptical shapes) as well as microholes (circular outlines) (Figure 3 and Figure 4), thus confirming that both the presence of cavities and their shapes were not instrumental artefacts. The origin of these cavities is unclear. The microholes might originate from gases released after heating, but we do not have an explanation for the mechanism from which the nanoholes originate. A speculative hypothesis is that the nanoholes are the morphological expression of a local partial order of Fe-POL-forming molecules, as suggested by the by X-ray diffraction pattern [14]_._ The perfect alignment of the nanoholes may support this hypothesis, but it is unknown how they formed.

**Figure 5:**
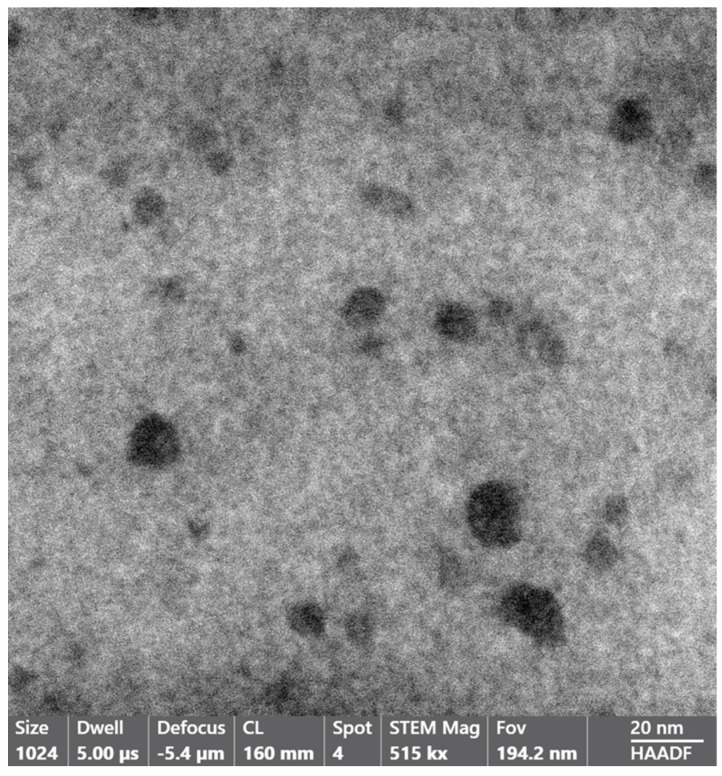
Image of Fe−POL (STEM mode). The size of the holes varies from 4 nm to 16 nm. The shape is not always circular or subcircular, but it can also be pseudopolygonal (cavity at the bottom right is an example).

**Figure 6:**
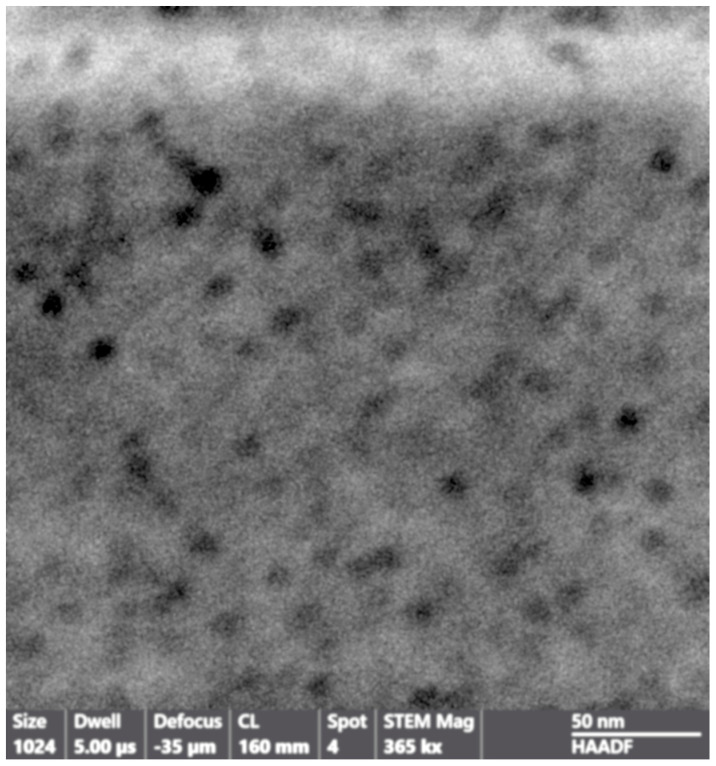
Image of Na−POL (STEM mode). The size of the holes in this picture is more frequently 4–5 nm. The shape is slightly elongated but also circular.

The dimensional inhomogeneity of the holes drew our attention not only for understanding their mechanism of formation but also for ascertaining if the cavities formed during the preparation of Fe-POL, and therefore, where the place in which iron, likely as hydroxide, precipitated to give origin to hematite after heating was. To this aim, internal portions of Fe-POL as well as Na-POL were observed and analysed by TEM and AEM. Both copolymers were characterized by the presence of nanoholes (Figure 5 and Figure 6). The hole sizes ranged from 4 nm to 20 nm, and the shapes were not uniform; they were circular, subcircular, or pseudopolygonal.

**Figure 7:**
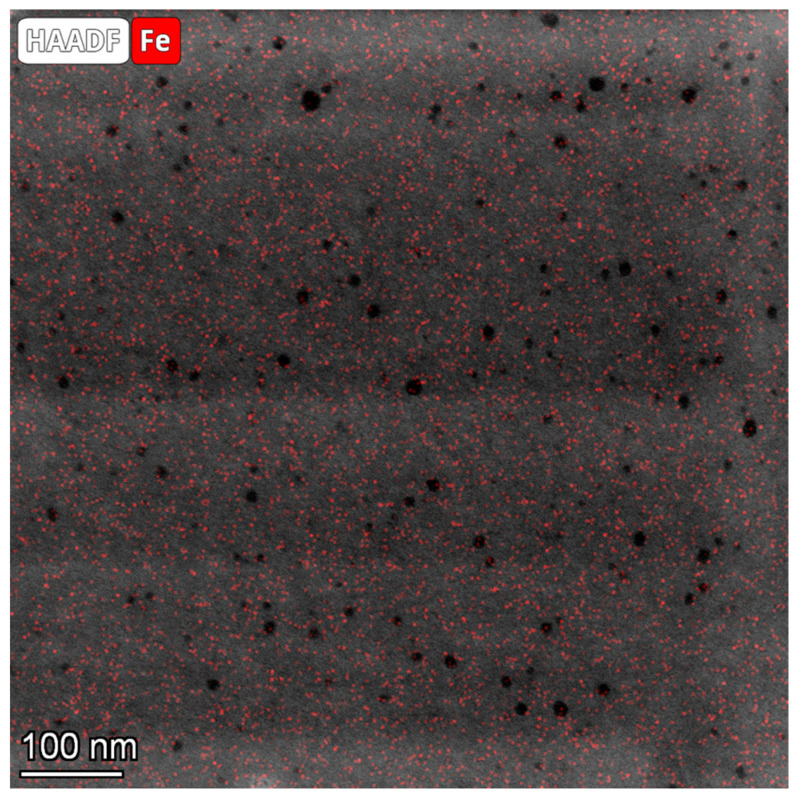
Fe-POL image obtained by overlapping an HAADF image with the iron map. The Fe signal of the nanoparticles appears as dots of a very small size distributed over the polymer surface, without any defined distribution pattern. The nanoparticles do not appear to form aggregates, nor are they preferentially located within the polymer cavities.

In the case of Fe_POL, the holes did not contain Fe hydroxides, as clearly shown in Figure 7 where the HAADF image and Fe map overlap. The size of the Fe-nanoparticle (4–6 nm) is smaller than those we estimated (mean value = 21 nm) by powder XRD [14] and image analyses. This is not surprising as the Scherrer equation [23] is known to provide only a rough estimation of particle sizes.

**Figure 8:**
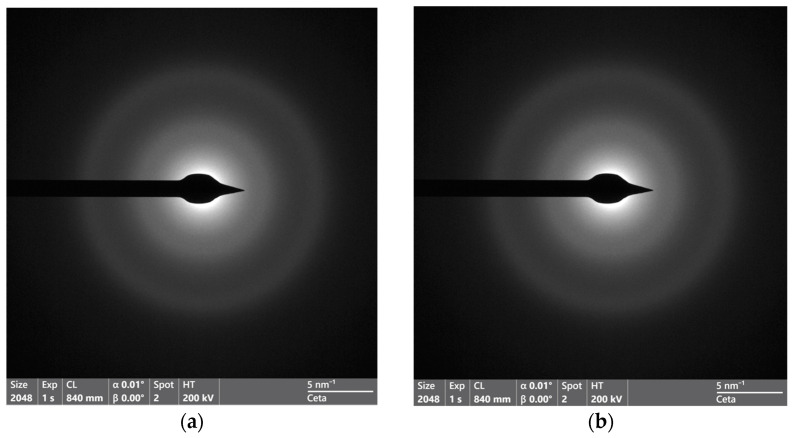
SAED patterns of Na−POL (**a**) and Fe−POL (**b**). The image of the Na−POL shows only two diffraction rings (lines at 0.20 and 0.11 nm), which indicates that the crystalline arrangement of the molecules forming the polymer is very low. On the other hand, the Fe−POL pattern does not show any difference with respect to Na−POL, and no additional diffraction signal associated with the possible presence of Fe nanoparticles is observed, confirming that Fe is present as (copolymerized) Fe(AAEMA)_3_.

SAED images of Na-POL and Fe-POL do not differ from one another (Figure 8a,b), thus implying that the two holes’ halos (at approximately 0.20 and 0.11 nm) are not due to metal hydroxides (that, as expected, are amorphous) but to the polymer molecules that are locally ordered.

## 4. Conclusions

This study highlights rather new physical properties of AAEMA^-^ copolymers: ordered porosity and proto-crystallinity.

To the best of our knowledge, these are the first microscopic observations documenting the existence of nanoholes, with elliptical shapes perfectly aligned in two directions, thus resembling a grid. They were observed only in some grains, together with larger pores with circular outlines. The circular pores might be formed from volatile compounds released after heating, but we do not have explanations about the formation of nanoholes.

After heating at 150 °C, Na and Fe AAEMA polymers are not amorphous as believed, but partially crystalline, thus suggesting that the order of polymer-forming molecules is induced by the temperature.

As previously believed, this is a research topic that should draw the attention of materials scientists; controlling the porosity and amorphous/crystalline state by changing the heating temperature can lead to various industrial applications of metal organic polymers.

## Data Availability

Data are contained within the article.
